# Pharmacological interventions for preventing postoperative nausea and vomiting in adult patients undergoing ambulatory surgery: Protocol for a systematic review and network meta-analysis

**DOI:** 10.1097/MD.0000000000016605

**Published:** 2019-07-19

**Authors:** Geun Joo Choi, Je Jin Lee, Hee-Kyeong Seong, Hyun Kang

**Affiliations:** Department of Anesthesiology and Pain Medicine, Chung-Ang University College of Medicine, Seoul, Republic of Korea.

**Keywords:** ambulatory anesthesia, nausea, network meta-analysis, systematic review, vomiting

## Abstract

**Background:**

We aimed to conduct a systematic review and network meta-analysis (NMA) of published studies to comprehensively compare and rank the efficacy and safety of pharmacological interventions for preventing nausea and vomiting after ambulatory surgery.

**Methods:**

A systematic and comprehensive search will be performed using the MEDLINE, EMBASE, the Cochrane Central Register of Controlled Trials, and Google Scholar databases, beginning from their inceptions to July and August 2019. Only randomized clinical trials on the efficacy and safety of pharmacologic interventions for preventing nausea and vomiting after ambulatory surgery will be included.

The primary endpoints will be the incidences of postoperative nausea (PON), postoperative vomiting (POV), and postoperative nausea and vomiting (PONV) in the following recovery phases: before discharge (recovery phase I and II), after discharge but within 24 hours following surgery, and after discharge, after the initial 24-hour postoperative period.

The incidences of delayed post-discharge nausea, post-discharge vomiting, and post-discharge nausea and vomiting, which occur after the initial 24-hour postoperative period, severities of PON, POV, and PONV, use of rescue antiemetics, and the incidence of complete response, as well as safety issues, including complications, such as headache, dizziness, and drowsiness, will be also assessed.

We will conduct both pairwise meta-analysis and NMA. We will use surface under the cumulative ranking curve values and rankograms to present the hierarchy of the pharmacologic interventions. A comparison-adjusted funnel plot will be used to assess the presence of small-study effects. The quality of the studies included will be assessed using the risk of bias tool 2.0. All statistical analyses will be performed using Stata SE, version 15.0 (StataCorp, College Station, TX).

**Results:**

The results of this systematic review and NMA will be published in a peer-reviewed journal.

**Conclusion:**

This systematic review and NMA will provide comprehensive and convincing evidence summarizing the efficacy and safety of pharmacological interventions for preventing nausea and vomiting after ambulatory surgery.

**Trial registration number:**

CRD42018103068.

## Abstract 2

Supplemental Digital Content is available in the text

## 1 Introduction

The recent advances in short-acting anesthetics, anesthetic techniques, and minimally invasive surgical techniques have led to a tremendous growth of ambulatory surgery. In the United States, 65% to 70% of all operations are performed in an outpatient setting.^[1]^ According to the development of medical science and techniques, ambulatory surgery will be a broad and expanding area of surgical practice.

Postoperative nausea and vomiting (PONV) after ambulatory surgery is a serious issue because it may result in a prolonged discharge or an unanticipated admission, both of which lead to an increase in the healthcare cost. Furthermore, nausea and vomiting may occur or recur after discharge when patients have limited access to effective treatment. Post-discharge nausea and vomiting (PDNV) occurs with an incidence of approximately 30%.^[2]^ Considering the above, the prevention of PONV, including PDNV, is of particular importance in ambulatory surgery.

PONV prophylaxis is one of the extensively studied areas. A number of studies have reported their results regarding many types of pharmacological interventions for PONV, including 5-hydroxytryptamine (5-HT_3_) receptor antagonists, neurokinin-1 (NK-1) receptor antagonists, dopamine (D_2_) receptor antagonist, corticosteroids, butyrophenones, antihistamines, anticholinergics, phenothiazines, and other drugs with an antiemetic effect. However, the relative efficacy and safety of these pharmacological interventions still remain unknown.

Therefore, we plan to conduct a systematic review and network meta-analysis (NMA) of published studies to comprehensively compare and rank the efficacy and safety of pharmacological interventions for preventing nausea and vomiting after ambulatory surgery.

## 2 Methods

### 2.1 Protocol design and registration

This protocol was developed following the preferred reporting items for systematic review and meta-analyses (PRISMA) extension statement for NMA.^[3]^ The protocol for this systematic review and NMA has been registered with the International Registration of Prospective Systematic Reviews (PROSPERO network; registration number CRD42018103068).

### 2.2 Eligibility criteria

#### 2.2.1 Type of studies

Peer-reviewed, randomized controlled trials (RCTs) will be eligible for inclusion. No language or date restriction will be applied. Review articles, case reports, case series, letters to the editor, commentaries, proceedings, laboratory science studies, and any other non-relevant studies will be excluded from this analysis.

#### 2.2.2 Participants

Adult participants undergoing ambulatory surgery under general anesthesia will be included. Those undergoing ambulatory surgery under regional anesthesia or sedation as anesthetic techniques will be excluded.

#### 2.2.3 Interventions and comparisons

Pharmacological interventions administered for the purpose of preventing nausea and vomiting after ambulatory surgery will be included. If interventions administered for other reasons can affect PONV, they will be included. The pharmacological interventions will belong to one of the following drug classes:

(1) 5-HT_3_ receptor antagonists;
(2) NK-1 receptor antagonists;
(3) D_2_ receptor antagonist;
(4) corticosteroids;
(5) butyrophenones;
(6) antihistamines;
(7) anticholinergics;
(8) phenothiazines; and
(9) other antiemetics.

We will include trials comparing 1 or more pharmacological intervention(s) for the prophylaxis of PONV against no treatment, placebo, or another pharmacological intervention(s). Prophylaxis means that the pharmacological interventions were administered before the participants experienced either nausea or vomiting. The timing of drug administration will be assessed as preoperative, intraoperative, and postoperative (before the occurrence of nausea or vomiting). Studies comparing nonpharmacological interventions, such as the administration of intravenous fluids, aromatherapy, or acupuncture, will be excluded.

#### 2.2.4 Outcomes

##### 2.2.4.1 Effectiveness

The primary endpoints will be the incidences of postoperative nausea (PON), postoperative vomiting (POV), and PONV at the following recovery phases:

(1) recovery phase before discharge (recovery phase I and II),
(2) recovery phase after discharge, within 24 hours following surgery,
(3) recovery phase after discharge, after the initial 24-hour postoperative period.

The outcomes of the recovery phase after discharge are considered as post-discharge nausea (PDN), post-discharge vomiting (PDV), and PDNV. We will evaluate the incidences of delayed PDN, PDV, and PDNV, which occur after the initial 24-hour postoperative period. The severities of PON, POV, and PONV, and the use of rescue antiemetics and the incidence of complete response will also be assessed.

##### 2.2.4.2 Safety

Safety issues, including complications, such as headache, dizziness, and drowsiness will be assessed. The frequency and reason of readmission will be identified.

### 2.3 Information sources

#### 2.3.1 Electronic search

A search will be performed in the MEDLINE, EMBASE, the Cochrane Central Register of Controlled Trials, and Google scholar databases using search terms related to PONV. The search strategy, which includes a combination of free text, Medical Subject Heading, and EMTREE terms, is outlined in the Supplemental Digital Content (Appendix).

Additional relevant articles will be identified by scanning the reference lists of articles found during the original search and meta-analyses. Reference lists will be imported into Endnote software (Thompson Reuter, CA), and duplicate articles will be removed.

#### 2.3.2 Study selection

The titles and abstracts identified through the search strategy described above will be scanned independently by 2 of the study's authors (GJC and JJL). To minimize data duplication as a result of multiple reporting, papers from the same author will be compared. For studies determined to be eligible based on the title or abstract, the full paper will be retrieved. All abstracts that cannot provide sufficient information regarding the eligibility criteria will be selected for full-text evaluation. Any potentially relevant studies chosen by at least one of the authors will be retrieved and their full-text versions will be evaluated. In the second phase, the same reviewers will independently evaluate the full-text articles and make their selection in accordance with the eligibility criteria.

The articles that will meet the inclusion criteria will be assessed separately by 2 of the study's authors (GJC and JJL), and any discrepancies will be resolved through a discussion. In cases where an agreement cannot be reached, the dispute will be resolved with the help of a third investigator. A flow diagram for the search and selection process that follows the PRISMA guidelines will be developed.

#### 2.3.3 Data extraction

All interrelated data from the included studies will be independently extracted and entered into a standardized form by 2 of the study's authors (GJC and HS) and will then be cross-checked. Any discrepancy will be resolved through a discussion. If an agreement cannot be reached, the dispute will be resolved with the aid of a third investigator (HK).

The standardized extraction form includes the following items, and the data will be extracted independently by 2 of the study's authors (GJC and HS):

(1) title;
(2) authors;
(3) name of journal;
(4) publication year;
(5) study design;
(6) registration of clinical trial registry;
(7) competing interests;
(8) country;
(9) risk of bias (ROB);
(10) number of patients in study;
(11) kinds and doses of drugs compared;
(12) sex of patients;
(13) age of patients;
(14) weight of patients;
(15) height of patients;
(16) duration of anesthesia;
(17) American Society of Anesthesiologists’ physical status score;
(18) inclusion criteria;
(19) exclusion criteria;
(20) type of surgery;
(21) type of anesthesia;
(22) incidences of PON, POV, and PONV before discharge (during recovery phase I and recovery phase II);
(23) incidences of PDN, PDV, and PDNV within 24 hours following surgery and after the initial 24 hours following surgery;
(24) severity of PON, POV, and PONV;
(25) the need for rescue antiemetics;
(26) number of cases of complete response; and
(27) number of cases of reported headache, dizziness, drowsiness, or constipation.

The postoperative period will be divided into 3 phases: before hospital discharge; after discharge, but within postoperative 24 hours; and after discharge, but after the initial 24 hours following surgery. The outcomes will be presented as PON, POV, and PONV; PDN, PDV, and PDNV; delayed PDN, PDV, and PDNV, respectively. If a study reported the data at multiple time points within the same phase, the data from the first time point will be extracted as the outcome of interest. If the data from the first time point was reported as 0 hour at a post-anesthesia care unit or immediately after surgery during recovery phase I, we will extract the second time point as the outcome of interest. If a study did not mention the outcomes as data after discharge, which are expected to be reported at a time point after discharge considering the patients’ discharge time, we will regard the data as outcomes after discharge, and extract them. The outcomes regarding safety issues will be collected as those before discharge and after discharge.

If information is missing, an attempt will be made to contact the study authors to obtain the relevant information. If data are presented as figures rather than numbers, the open-source software Plot Digitizer (version 2.6.8; http://plotdigitizer.sourceforge.net) will be used to extract the numbers. For studies reporting the results from different doses in the same study, the groups will be combined in order to avoid a unit of analysis error.

The degree of agreement between the 2 independent data extractors will be computed using kappa statistics to measure the difference between the observed and expected agreements; namely, whether they were random or by chance only. Kappa values will be interpreted as follows:

(1) less than 0: less than chance agreement;
a. to 0.20: slight agreement;
(2) 0.21 to 0.40: fair agreement;
(3) 0.41 to 0.60: moderate agreement;
(4) 0.61 to 0.80: substantial agreement; and
(5) 0.8 to 0.99: almost perfect agreement.^[4]^

### 2.4 Study quality assessment

The quality of the studies will be independently assessed by 2 of the study's authors (GJC and HK), using the Revised Cochrane ROB tool for randomized trials (RoB 2.0).^[5]^ The ROB will be evaluated by considering the following 5 potential sources of bias:

(1) bias arising from the randomization process;
(2) bias due to deviations from the intended interventions;
(3) bias due to missing outcome data;
(4) bias in measurement of the outcome; and
(5) bias in selection of the reported result.

Then, we will evaluate the overall ROB judgment according to these domain-level judgments. The methodology for each domain will be graded as “Low ROB,” “Some concerns,” and “High ROB,” which reflect a low ROB, some concerns, and a high ROB, respectively.^[5]^

### 2.5 Statistical analysis

Ad-hoc tables will be designed to summarize the data from the included studies and show their key characteristics and any important questions related to the aim of this review. If a trial result is presented with 0 events in 1 group, then the event rate will be artificially inflated by adding 0.5. After the data have been extracted, reviewers will determine whether a meta-analysis is possible. For this, we will evaluate the heterogeneity and transitivity assumptions by examining the comparability of the patients’ eligibility criteria, pertinent patients’ demographics, study design, and the ROB (all degrees of bias versus removing a “High ROB” arising from the randomization process and bias in measurement of the outcome) as potential treatment-effect modifiers across comparisons.^[6]^ We will note the methodological differences between the studies that could influence the outcome measurement, as well as any concerns related to the transitivity assumption or methodological heterogeneity.

Both a standard pairwise meta-analysis and a NMA will be conducted.

Initially, when at least 2 studies examine the same drugs, a pairwise meta-analysis will be conducted to generate summary estimates and to assess the statistical heterogeneity across the included studies. The summary estimates will be reported as mean differences, standardized mean differences, or relative risks (RRs), as appropriate, with corresponding 95% confidence intervals (CIs). The heterogeneity between the studies will be assessed using the Cochran *Q* and the Higgins *I*^2^ statistics. A level of 10% significance (*P* < .10) in the Chi^2^ statistic or an *I*^2^ greater than 50% will be regarded as considerable heterogeneity, and the data will be analyzed using the Mantel-Haenszel random-effect model. Otherwise, the Mantel–Haenszel fixed-effect model will be applied.^[7]^

Publication bias will be assessed using the Begg funnel plot and the Egger test. If the funnel plot is asymmetrical or the *P*-value is found to be <.1 by the Egger test, the presence of a publication bias will be considered, and trim and fill analyses will be performed.

When the treatment nodes form a connected network of evidence, we will perform a NMA. A multiple treatment comparison NMA is a generalization of meta-analysis methods that include both the direct RCT comparisons as well as the indirect comparisons of treatments. An NMA based on a frequentist framework will be performed using the NMA graphical tools by Chaimani et al.^[8]^ Given the clinical and methodological heterogeneity of the populations and methods among the included trials in the NMAs, we will use the random-effects model in our primary analyses.

A network plot linking all the included analgesics will be formed to indicate the type of analgesics, the number of patients under different analgesics, and the amount of pair-wise comparisons. In the network plot, nodes will show the analgesic being compared, and edges will show the available direct comparisons between the analgesics. Each drug, as well as each combination of drugs, will be treated as a node in this network. Nodes and edges will be weighted according to the number of patients and studies, respectively.

We will examine the consistency of the total network through both global and local tests of inconsistency. We will evaluate the global consistency assumption using the design-by-treatment interaction model.^[9]^ We will also evaluate each closed loop in the network in order to examine the local inconsistency between the direct and indirect effect estimates for the same comparison. In each loop, we will estimate the inconsistency factor (IF) as the absolute difference (with 95% CIs and a *z*-test) between the direct and indirect estimates for each paired comparison in the loop. The IF is the logarithm of the ratio of 2 odds ratios (RoR) from the direct and indirect evidence in the loop; RoR values close to 1 indicate that the 2 sources are in agreement.

We will also show the relative treatment effects between all active medications in ranked forest plots. The mean summary effects with CIs will be presented together with their predictive intervals (PrIs) to facilitate the interpretation of the results in light of the magnitude of heterogeneity. PrIs provide an interval that is expected to encompass the estimate of a future study. We will not adjust for multiple comparisons in successive NMAs, as we are not interested in establishing the superiority or inferiority of particular comparisons.

A rankogram and cumulative ranking curve will be drawn for each analgesic. A rankogram plots the probabilities for treatments to assume any of the possible ranks. It is the probability that a given treatment ranks first, second, third, and so on, among all of the treatments evaluated in the NMA. We will use the surface under the cumulative ranking curve (SUCRA) values to present the hierarchy of the interventions. SUCRA is a relative ranking measure that accounts for the uncertainty in the treatment order, meaning it accounts for both the location and the variance of all relative treatment effects.^[10]^ A higher SUCRA value is regarded as a better result for an individual intervention. When ranking treatments, the closer the SUCRA value is to 100%, the higher the treatment ranking is, relative to all other treatments.

We will test for small-study effects and publication bias using the comparison-adjusted funnel plot.^[11]^

A standard pairwise meta-analysis and NMA will first be performed based on the data derived purely from studies for each drug, or for combinations of drugs, and re-analyzed according to the study design.

If clinical and methodological heterogeneity between the study arms is found to be substantial, we will present the pairwise meta-analysis only. If the transitivity assumption cannot adequately be met, a descriptive summary of the study findings will be presented. If an inconsistency in the entire network or a local inconsistency is suspected, we will conduct sensitivity analyses to evaluate the reason for the inconsistency, as well as the influence of individual studies on the overall effect estimate by excluding 1 study at a time from the analysis. All statistical analyses will be performed using Stata SE, version 15.0 (StataCorp, College Station, TX).

## 3 Evidence synthesis

Based on the results of the NMA for the RCTs, the overall quality of evidence for each outcome assessed will be rated using the guidelines developed by the grading of recommendations assessment, development, and evaluation working group. These guidelines are designed to rate the quality of the effect estimates derived from an NMA and uses a sequential assessment of the evidence quality, followed by an assessment of the risk-benefit balance and a subsequent judgment on the strength of the recommendations.^[12]^ We will use a 4-step process:

(1) present direct and indirect treatment estimates (mean differences, standardized mean differences, or RRs with 95% CIs);
(2) rate the quality of the direct and indirect treatment estimates;
(3) present the NMA estimates (pool of direct and indirect estimates, mean differences, standardized mean differences, or RRs with 95% CIs); and
(4) rate the quality of the NMA estimates.

## 4 Ethics and dissemination

### 4.1 Ethical issues

This systematic review does not require ethics approval or patients’ informed consent because there will be no direct contact with individual patients. Only previously published data will be included in the review.

### 4.2 Publication plan

This systematic review will be published in a peer-reviewed journal and will be disseminated electronically and in print.

## 5 Discussion

PONV is the main concern in the patient management after surgery. It occurs with an overall average incidence of about 30%, considering all patient and surgery types.^[13]^ In an ambulatory setting, the patient management system is different compared to that of inpatients. The healthcare environment requires that patients are quickly and efficiently shifted through the system from admission to discharge. During this process, less adverse events during the recovery phase may be associated with the possibility of discharge on the day of surgery. Further, we should be able to manage the patients undergoing ambulatory surgery considering their condition during the post-discharge period. In the ambulatory population, nausea and vomiting may occur or recur following discharge when patients have a limited access to an effective treatment. PDNV occurs with an incidence of approximately 30%.^[2,14]^ The incidence of PONV has been demonstrated to be as high as 80% in high-risk groups, such as patients undergoing laparoscopy, tonsillectomy, or strabismus repair, which can be performed in an ambulatory setting, with no prophylactic antiemetic therapy.^[13,15]^ Thus, the prevention of PONV is a crucial problem in the patients undergoing ambulatory surgery, and we should apply appropriate pharmacological interventions for the prophylactic strategy.

The purpose of the present study is to provide a clinically useful ranking of pharmacological interventions for PONV prophylaxis following ambulatory surgery, as well as to provide evidence for physicians that will guide them towards clinical decisions that enhance the efficacy and safety.

## Author contributions

**Conceptualization:** Hee-Kyeong Seong.

**Data curation:** Je Jin Lee, Hyun Kang.

**Funding acquisition:** Hyun Kang.

**Investigation:** Geun Joo Choi, Je Jin Lee.

**Methodology:** Geun Joo Choi, Hee-Kyeong Seong.

**Project administration:** Hyun Kang.

**Writing – original draft:** Geun Joo Choi, Je Jin Lee, Hee-Kyeong Seong.

**Writing – review and editing:** Hyun Kang.

Hyun Kang orcid: 0000-0003-2844-5880.

## Supplementary Material

Supplemental Digital Content
