# Supplementary material for: Pharmacological interventions for preventing postoperative nausea and vomiting in adult patients undergoing ambulatory surgery: Protocol for a systematic review and network meta-analysis
Source: Medicine (Baltimore). 2019 Jul 19;98(29):e16605. doi: 10.1097/MD.0000000000016605 (PMC6708945; doi:10.1097/MD.0000000000016605)
Supplement: Supplemental Digital Content [file medi-98-e16605-s001.docx]

**Search terms for MEDLINE**

1. randomized controlled trial.pt
2. randomized controlled trial$.mp
3. controlled clinical trial.pt
4. controlled clinical trial$.mp
5. random allocation.mp
6. exp double-blind method/
7. double-blind.mp
8. exp single-blind method/
9. single-blind.mp
10. or/1-9
11. clinical trial.pt
12. clinical trial$.mp
13. exp clinical trial/
14. (clin$ adj25 trial$).mp
15. ((singl$ or doubl$ or tripl$ or trebl$) adj25 (blind$ or mask$)).mp
16. random$.mp
17. exp research design/
18. research design.mp
19. or/11-18
20. 10 or 19
21. Case report.tw.
22. Letter.pt.
23. Historical article.pt.
24. Review.pt.
25. or/21-24
26. 20 not 25
27. Exp Ambulatory Surgical Procedures/
28. Exp outpatients/
29. ambulatory surgery.mp
30. ambulatory anesthesia.mp
31. one day surgery.mp
32. one-day surgery.mp
33. day surgery.mp
34. outpatient surgery.mp
35. outpatient anesthesia.mp
36. ambulatory intervention.mp
37. ambulatory procedure.mp
38. outpatient intervention.mp
39. outpatient procedure.mp
40. ambulatory anaesthesia.mp
41. outpatient anaesthesia.mp
42. or/27-41
43. 26 and 42
44. Exp nausea/
45. Exp nausea and vomiting/
46. Nausea.mp
47. Exp vomiting/
48. Vomit.mp
49. Retching.mp
50. Retch.mp
51. Emesis.mp
52. Emetic.mp
53. PONV.mp
54. PDNV.mp
55. Exp Antiemetics/
56. Or/44-55
57. 43 and 56

**Search terms for Embase**

1. randomi?ed controlled trial$.mp.
2. 'controlled clinical trial (topic)'/exp
3. controlled AND clinical AND trials
4. controlled clinical trial$.mp.
5. 'randomization'/exp
6. 'random allocation'/exp
7. random allocation.mp.
8. double-blind.mp.
9. single-blind.mp.
10. #1 OR #2 OR #3 OR #4 OR #5 OR #6 OR #7 OR #8 OR #9
11. 'clinical trial (topic)'/exp
12. clinical AND trial$.mp.
13. random$.mp.
14. rct
15. #11 OR #12 OR #13 OR #14
16. #10 OR #15
17. 'case study'/exp
18. 'case report'/exp
19. 'abstract report'/exp
20. 'letter'/exp
21. #17 OR #18 OR #19 OR #20
22. #16 NOT #21
23. 'ambulatory surgery'/exp
24. 'outpatient'/exp
25. 'ambulatory surgery'
26. 'ambulatory anesthesia'
27. ‘one day surgery’
28. ‘one-day surgery’
29. ‘day surgery’
30. ‘outpatient surgery’
31. ‘outpatient anesthesia’
32. ‘ambulatory intervention’
33. ‘ambulatory procedure’
34. ‘outpatient intervention’
35. ‘outpatient procedure’
36. ‘ambulatory anaesthesia’
37. ‘outpatient anaesthesia’
38. ‘outpatient anaesthesia’
39. #23 OR #24 OR #26 OR #27 OR #28 OR #29 OR #30 OR #31 OR #32 OR #33 OR #34 OR #35 OR #36 OR #37 OR #38
40. #22 AND #39
41. 'nausea'/exp
42. 'nausea and vomiting'/exp
43. 'postoperative nausea and vomiting'/exp
44. Nausea
45. 'vomiting'/exp
46. Vomit
47. Retching
48. Retch
49. Emesis
50. Emetic
51. PONV
52. PDNV
53. 'antiemetic agent'/exp
54. #41 OR #42 OR #43 OR #44 OR #45 OR #46 OR #47 OR #48 OR #49 OR #50 OR #51 OR #52 OR #53
55. #40 AND #54
